# Deciphering the Role Played by Autophagy in *Leishmania* Infection

**DOI:** 10.3389/fimmu.2019.02523

**Published:** 2019-11-01

**Authors:** Patricia Sampaio Tavares Veras, Juliana Perrone Bezerra de Menezes, Beatriz Rocha Simões Dias

**Affiliations:** ^1^Laboratory of Host – Parasite Interaction and Epidemiology, Gonçalo Moniz Institute, Salvador, Brazil; ^2^National Institute of Science and Technology of Tropical Diseases - CNPq, Salvador, Brazil

**Keywords:** autophagy, *Leishmania*, LC3, macrophages, phagocytosis, leishmaniasis, parasitophorous vacuoles

## Abstract

In recent decades, studies have shown that, depending on parasite species and host background, autophagy can either favor infection or promote parasite clearance. To date, relatively few studies have attempted to assess the role played by autophagy in *Leishmania* infection. While it has been consistently shown that *Leishmania* spp. induce autophagy in a variety of cell types, published results regarding the effects of autophagic modulation on *Leishmania* survival are contradictory. The present review, after a short overview of the general aspects of autophagy, aims to summarize the current body of knowledge surrounding how *Leishmania* spp. adaptively interact with macrophages, the host cells mainly involved in controlling leishmaniasis. We then explore the scarce studies that have investigated interactions between these parasite species and the autophagic pathway, and finally present a critical perspective on how autophagy influences infection outcome.

## Introduction

Originally described by Christian de Duve in mammalian systems in 1963 (1), autophagy was first viewed as a selective sequestration process thought to occur as a result of the engulfment of cytosolic senescent material (2–4). Knowledge surrounding the molecular mechanisms underlying autophagy would only develop in the following decades, paved by genetic studies performed in yeast, which unveiled more than 30 proteins, denominated as Atg proteins, linked to autophagosome formation (5, 6). Thereafter, cumulative studies connected this catabolic pathway to the degradation of superfluous and damaged cytosolic material or organelles, resulting in the recycling of macromolecular constituents for reuse by cellular machinery, thereby promoting the maintenance of cellular homeostasis (7, 8).

Autophagy participates in a variety of physiological processes, such as the generation of amino acids under starvation conditions, the quality control of intracellular proteins and organelles, the regulation of expression levels of selective substrates, the degradation of pathogens and antigen presentation, all of which have been recently analyzed by several comprehensive reviews (9–16). Subsequently, this collective body of evidence would lead researchers to conduct a variety of studies that effectively associated autophagy with disease conditions, including neurodegenerative disorders (17) and pathogen infection (18–21), among others (22, 23).

In response to microbe infections, mammalian cells can activate autophagy that can either cause parasite destruction or result in pathogen survival. For parasites of the *Leishmania* genus, the role played by autophagy in the context of infection remains not well-understood. This review aims to describe the studies that have explored interactions between these parasite species and the autophagic pathway, as well as present a critical perspective on how autophagy influences infection outcome.

## General Aspects of Autophagy

**Figure 1:**
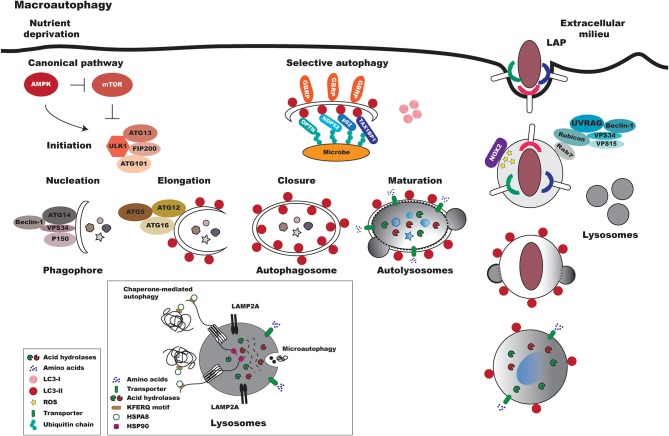
Overview of canonical and non-canonical mammalian autophagy processes. In response to reduced nutrient availability, the AMPK protein is activated, leading to the repression of mTOR. The ULK1-ATG13-FIP200-ATG101 complex is then activated, which triggers the autophagic pathway. Macroautophagy can be divided into a series of coordinated and consecutive events. In the first stage, denominated nucleation, proteins are recruited to form the phagophore, a double-membraned structure. The main proteins involved in this early stage of autophagosome formation are VPS34, Beclin-1, Atg14, and p150. The expansion/elongation of the phagophore occurs concurrently with the nucleation process. Two ubiquitin-like conjugation systems are involved in the expansion of the phagophore: the Atg12-Atg5-Atg16 and LC3 systems. Lastly, autophagosomes fuse with lysosomes to became autophagolysosomes, in which acid hydrolases degrade the sequestered materials and released the degraded products into the cytoplasm. In xenophagy, intracellular pathogens are ubiquitinated and recognized by autophagic adapters (e.g., OPTN, NDP52, p62, TAX1BP1). These adapters then deliver pathogens to autophagosomes by directly binding with LC3-II. As in macroautophagy, the autophagosomes fuse with lysosomes to form autolysosomes. Pattern recognition receptors (e.g., TLRs, Fc receptors, and CLEC7A/dectin-1) can trigger LAP. In this process, Rubicon associates with the PI3K class 3 complex, formed by VPS34, VPS15, UVRAG (UV radiation resistance-associated gene), and Beclin-1, resulting in the stabilization of NOX2. Subsequently, reactive oxygen species (ROS) are generated by NOX2, leading to LC3 recruitment to single-membrane vacuoles. Finally, the LAPosome fuses with lysosomes. In microautophagy, cytoplasmic components are directly engulfed by the lysosomal membrane. In chaperone-mediated autophagy, chaperones recognize soluble proteins with pentapeptide motifs (KFERQ) and deliver them to lysosomes for degradation.

In mammalian cells, regardless of the method of pathway activation, three primary types of autophagy have been identified: chaperone-mediated autophagy, microautophagy and macroautophagy, all of which culminate in the delivery of engulfed cargo material to lysosomes to complete degradation and recycling [Figure 1; (24)]. As recent reviews have already comprehensively discussed these processes, this text will not endeavor to offer any further elucidation (1, 25, 26).

Macroautophagy, generally referred to as autophagy or canonical autophagy, is the most important type of autophagy, and has thusly been widely analyzed. Compared to other cellular vesicle-mediated transport processes, macroautophagy is a unique vesicular process that, in response to several types of stimuli, culminates in the formation of the autophagosome (Figure 1). Differently from other membrane-bound organelles formed through membrane budding via a pre-existing compartment, this vesicle is formed *de novo* by coordinated events orchestrated by Atg proteins. In general, autophagosome formation initiates through nucleation and is followed by elongation and closure processes before reaching maturation, in which the autolysosome is formed through fusion with lysosomes [Figure 1; (1, 25, 27)].

In contrast to non-selective autophagy, selective autophagy encompasses events that first involve the ubiquitination of cytosolic cargos that are recognized via receptors in adaptor molecules, known as cargo receptors that recognize LC3/GABARAP family members [Figure 1; (28–39)]. Similarly to the non-selective autophagy pathway, the material engulfed by the selective process is transported to autolysosomes for degradation (26, 40).

More recently, a non-canonical form of autophagy has been described, denominated as LC3-associated phagocytosis (LAP) (Figure 1). In contrast to other autophagic pathways, the LAP process involves the recruitment of the LC3-phosphatidylethanolamine (PE) conjugation system, which is required for lysosomal fusion and maturation of the LAPosome, resulting in the engulfment of living or non-living particles. LAP is considered an interconnecting pathway between autophagy and phagocytosis and, similar to this latter pathway, was primarily described as a degradative pathway responsible for the control of pathogen proliferation (41). Similarly to what occurs in phagocytosis, pathogens can alternatively subvert the LAPosome pathway, thereby facilitating intracellular survival (42).

## Subversion of the Autophagic Pathway in Pathogen Infection

Autophagy as a host defense mechanism can act directly against pathogens through elimination within autophagosome compartments, or by indirectly facilitating infection through the modulation of signaling pathways involved in innate and adaptive immune responses (43–46). In this context, autophagy has been demonstrated as one of the most important mechanisms described in the last two decades that advantageously facilitates pathogen intracellular survival by diverting normal phagosomal trafficking, since microbes are redirected from phagosomes to autophagosomes following take-up by mammalian cells, including *C. burnetti* and *L. pneumophila* (47–49). In contrast, for other types of pathogens, such as *Listeria monocytogenes* and *Mycobacterium tuberculosis*, the autophagosome-like compartment induced by these microbes seems to present toxicity, which triggers some pathogens to escape into the cytoplasm of mammalian cells. In this escape mechanism, a variety of intracellular microbes modulate the autophagic pathway on a molecular level, allowing for parasite replication within host cells, thereby establishing persistent infection (50–53). Regarding the role of autophagy in *Leishmania* infection, a pioneering study published by Schaible et al. (54) reported that *L. mexicana*-parasitophorous vacuoles acquire macromolecules from the host cell cytoplasm by way of microautophagy. More recent studies attempting to investigate the role of autophagy in *Leishmania* infection produced contradictory results that do not clarify whether the exogenous induction of this pathway favors *Leishmania* survival or functions as a host defense mechanism (55–59).

## *Leishmania*-macrophage Interaction

*Leishmania* are inoculated by the sand fly vector during bloodfeeding and become rapidly phagocytized, predominantly by macrophages. Once inside these cells, surviving parasite promastigotes differentiate into amastigotes within phagolysosomal compartments, in which they are able to survive and proliferate. Thus, macrophages play an essential role in the establishment of infection and the persistence of parasites inside the mammalian host (60, 61).

During the initial interaction between parasites and macrophages, different species of *Leishmania* are recognized by a variety of macrophage receptors, including complement (CRs), Fcγ (FcγRs), fibronectin, and mannose receptors (MR). The recognition of the parasite by different receptors may impact the fate of intracellular parasites as well as the course of infection. Therefore, it is highly likely that, during natural infection, *Leishmania* are recognized simultaneously by more than one host cell receptor, and that specific combinations of these receptors result in differential activation that distinctively contributes to intracellular parasite survival (62–68).

The recognition of *Leishmania* parasites mainly via CR3 and CR1 inhibits inflammation and oxidative bursting, in addition to leading to the accumulation of LAMP1 and Cathepsin D in parasitophorous vacuoles (PVs). A study investigating CR3 recognition found that this receptor was associated with the uptake of metacyclic parasites, a more infective form of *Leishmania* (69). These authors also found that the mannose receptor, in combination with CR3, is associated with the uptake of avirulent promastigotes. Another study found that the presence of the CR3 cluster in caveolin and cholesterol-containing microdomains leads to delayed lysosome fusion, thusly favoring the replication of parasites within PVs (70). Together, these data show that *Leishmania* uptake via CR3 recognition could support the intracellular survival of these parasite species. On the other hand, the activation of complement receptors together with fibronectin receptors was shown to lead to an inflammatory response, thereby reducing parasite survival (68, 71). It was also demonstrated that *Leishmania* parasites degrade fibronectin in a GP63-dependent manner (72). The uptake of parasites via mannose receptor recognition may also trigger an inflammatory response by host cells, as well as provide more efficient delivery of hydrolytic enzymes into the macrophage phagolysosome (68). On the other hand, FcγR-mediated phagocytosis in bone marrow-derived macrophages (BMDM) was shown to promote IL-10 expression, which favors parasite survival and replication (73).

When promastigotes are recognized at the host plasma membrane, focal exocytosis, of macrophagic membranes from the endoplasmic reticulum, endosomes, and lysosomes, contributes to the formation of phagosomes containing *Leishmania* (74–76). Within these vacuoles with phagolysosomal features (77, 78), *Leishmania* promastigotes undergo a rapid transformation from metacyclic promastigotes, the infectious-stage form, into amastigotes (78). This differentiation process seems to be triggered by environmental changes, such as increases in temperature or decreased pH within the PV. Also, iron uptake and subsequent reactive oxygen species production by *Leishmania amazonensis* have been shown to play essential roles in parasite differentiation (79–82). While different species of *Leishmania* parasites all differentiate into amastigotes inside PVs, the formation of these vacuoles presents distinct dynamics and variable morphological features. Studies investigating these differences have highlighted complexities in PV formation (83, 84). Additionally, the PVs induced by *Leishmania* spp. can interact differently with a myriad of host-derived vesicles, including autophagic vesicles, which may have some influence on infection outcome (59).

## Autophagy in *Leishmania* Infection

### Autophagy and the Phagocytosis of *Leishmania*

**Figure 2:**
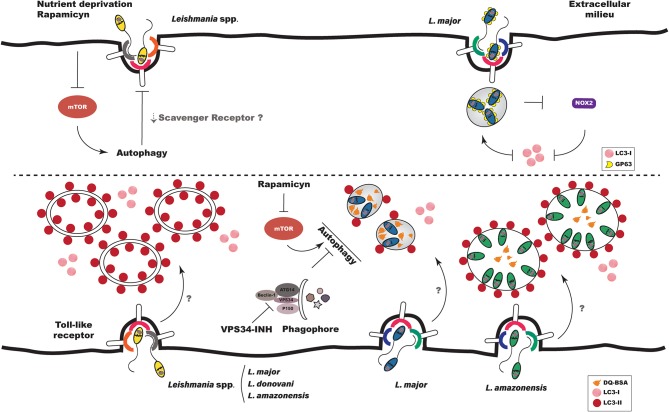
Autophagy in *Leishmania* infection. Interaction between *Leishmania* and the autophagic pathway occurs at different stages of infection. **(Top Left)** When autophagy is induced exogenously prior to infection, either by physiological or pharmacological means, the phagocytosis of *Leishmania* spp. is diminished, which could be related to decreases in scavenger receptors on host cell surfaces. **(Top Right)** *L. major* promastigotes evade LAP by inhibiting the recruitment of NOX2 and LC3 to the phagosomal membrane. **(Bottom Left)** *Leishmania* spp. induce autophagy in host cells both *in vitro* and *in vivo*. *L. major* parasites induce autophagy in BMDM by a mechanism dependent on Toll-like receptor 3. **(Bottom Right)** The parasitophorous vacuoles induced by *L. amazonensis* and *L. major* present distinct interaction with autophagic vacuoles. PVs induced by *L. major* are more degradative, while those induced by *L. amazonensis* recruit more LC3. LC3 recruitment to *L. major*- and *L. amazonensis*-induced PVs is not altered by either autophagic inhibition or induction.

Studies have demonstrated that the phagocytic pathway can communicate with the autophagic pathway, and that this communication enhances the microbicidal mechanisms involved in innate host immune response (85–87). In the context of *Leishmania* infection, the induction of autophagy, by either physiological (starvation) means or pharmacological treatment (e.g., with rapamycin, an mTOR inhibitor), was shown to inhibit the phagocytic ability of macrophages to engulf live *L. amazonensis* parasites, in addition to other large particles, such as latex beads, zymosan and yeast [Figure 2; (88)]. Using a model of *M. tuberculosis* infection, Bonilla et al. (89) demonstrated that the inhibition of autophagy favors the phagocytosis of this bacterium by C57BL/6 murine BMDM, which corroborated previous findings (88). These authors also demonstrated that the increased phagocytosis of *M. tuberculosis* by Atg 7 knockout macrophages was associated with higher expression of scavenger receptors MARCO (macrophage receptor with collagenous structure) and MSR1 (macrophage scavenger receptor 1). Interestingly, around a decade ago, the MARCO receptor was shown to be involved in the recognition of *Leishmania major* by murine macrophages (90). In sum, this evidence seems to indicate that the induction of autophagy negatively influences the general phagocytic capacity of macrophages, which could hypothetically be associated with scavenger receptors on the host cell surface (Figure 2).

Previously, Crauwels et al. (91) demonstrated, via a process involving LAP, that apoptotic *L. major* promastigotes recruited 13 times more LC3 to phagosomes than viable *L. major* promastigotes as early as 3 h after infection. This corroborated results presented by Matte et al. (92), which described the presence of LC3 labeling in only 10% of phagosomes containing WT *L. major* promastigotes after 1 h of infection. LC3 recruitment to phagosomes was also shown to be dependent on NOX2 activity in the context of LAP, since infection with Δgp63 parasites doubled the recruitment of LC3 to phagosomes. In addition, under the inhibition of NOX2 by DPI treatment, LC3 labeling in phagosomes containing Δgp63 parasites was reduced to levels similar to those containing WT parasites (92). Taken together, these findings suggest that, at least in the case of *L. major*, gp63 promastigote activity inhibits the migration of NOX2 to the phagosomal membrane, resulting in parasite escape from LAP-promoted engulfment, which could contribute to enhanced intracellular survival (Figure 2).

### Induction of Autophagy by *Leishmania* spp.

**Figure 3:**
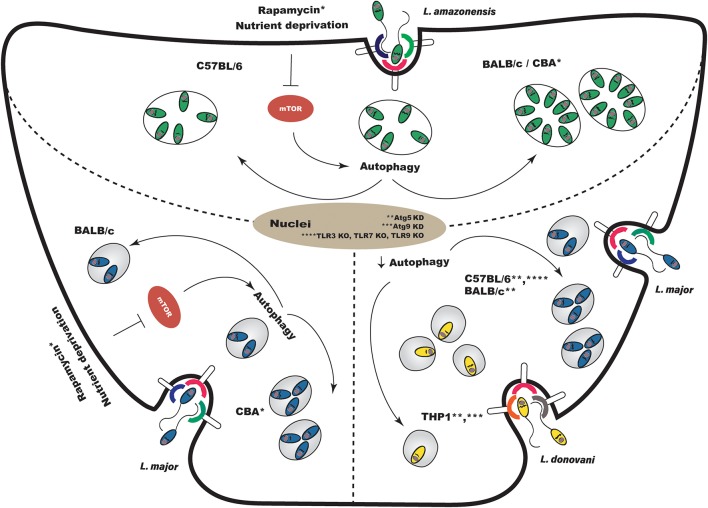
Effects of autophagic modulation on *Leishmania* infection outcome. **(Top)** Induction of autophagy following experimental *L. amazonensis* infection enhances parasite intracellular viability in susceptible BALB/c and CBA macrophages, but does not alter survival in resistant C57BL/6. **(Bottom Left)** Regarding *L. major*, the modulation of autophagy after infection increases intracellular parasite viability in CBA macrophages, but does not affect viability in BALB/c macrophages. **(Bottom Right)** Autophagic modulation using a genetic approach also leads to inconclusive results. Atg5 knockdown prior to infection in C57BL/6 and BALB/c macrophages enhanced *L. major* parasitic load. However, the knockdown, prior to infection, of Atg5 and Atg9 in THP-1 cells reduced *L. donovani* survival. In Tlr3/7/9 knockout C57BL/6 mouse macrophages, in which autophagy is not observed, *L. major* infection is not controlled. KD, knockdown; KO, knockout.

Several methods have been used to monitor the activation of autophagy in eukaryotic cells. In general, the confirmation of autophagic induction involves two or more methods, including transmission electron microscopy (TEM), quantification and detection of LC3, or the expression of other autophagy-related genes (Atg) (93). Most researchers investigating autophagic induction in macrophages subsequent to *Leishmania* infection have employed LC3 labeling by Western-blot or immunofluorescence (56–59, 92, 94–96). Using Western-blot, Cyrino et al. (56) detected LC3 labeling in extracts of *L. amazonensis*-infected macrophages from susceptible BALB/c and resistant C57BL/6 mice, as well as in the *L. amazonensis*-infected RAW 264.7 macrophage cell line (56). In addition, these authors found that LC3 labeling was positively correlated with parasitic load (56). Although Cyrino et al. (56) did not evaluate autophagic flux by treating infected cells with compounds that inhibit the autophagosome maturation process into autolysosomes, such as chloroquine, NH_4_Cl or bafilomycin, they nonetheless concluded that autophagy was indeed induced in *L. amazonensis*-infected macrophages. More recently, Frank et al. (95) showed, in BMDM from susceptible BALB/c mice, that *L. major* induces morphological alterations suggestive of autophagy, including the presence of myelin figures, cell vacuolization and double-membrane vesicles, all of which were observed by TEM. These authors also showed that *L. major* infection increased the ratio of LC3-II to LC3-I, reinforcing their morphological findings suggestive of autophagic pathway activation subsequent to *L. major* infection (95). This increase in the LC3-II to LC3-I ratio in response to *L. major* infection seems to occur independently of gp63, since similar ratios were observed in the extracts of macrophages infected with WT and Δgp63 promastigotes (92). More recent work elegantly demonstrated that *L. major* induces autophagy in BMDM of resistant C57BL/6 mice by way of a mechanism dependent on Toll-like receptor, since autophagy was not observed in Tlr3/7/9 knockout mouse macrophages, and these cells were not capable of controlling infection [Figure 3; (57)]. Another study showed that *Leishmania donovani* alternatively activated the autophagic pathway, as evidenced by higher LC3-II to LC3-I ratios detected in the infected human macrophage THP1 cell-line (58). Our group comparatively evaluated autophagic activation in macrophages using the CBA mouse model, as these animals are known to control *L. major*, yet are permissive to *L. amazonensis* infection. We found similar increases in the LC3-II/Actin ratio in the extracts of *L. major*- and *L. amazonensis*-infected macrophages (59). Consistent with findings reported in *in vitro* studies, Mitroulis et al. (94) observed greater LC3-I to LC3-II conversion in a sample of bone marrow macrophages from a male patient with visceral leishmaniasis arising from *L. donovani* infection, in comparison to a bone marrow sample from a healthy patient. Very recently, Pitale et al. (96) demonstrated that *L. donovani* not only induces autophagy in macrophages, but also in human polymorphonuclear neutrophils (PMNs). Additionally, higher numbers of LC3-labeled cells were detected in glomeruli samples from dogs naturally infected with *Leishmania infantum* as compared to samples from control animals (97). In sum, these findings suggest that, regardless of parasite species, *Leishmania* infection results in the activation of the autophagic pathway in host cells both *in vitro* and *in vivo* (Figure 2). Although the specific mechanisms by which autophagy is induced in host cells remain to be elucidated, preliminary evidence seems to point to activation being dependent on parasite species and host background.

Very few studies have addressed the timing of autophagy with regard to the establishment of *Leishmania* spp. infection. In the case of *L. donovani*, Pitale et al. (96) have demonstrated that while parasite induces non-canonical autophagy in PMNs at very early stages of infection, canonical autophagy was observed at later times. In THP-1 cells, it was demonstrated that although infection by *L. donovani* induces the activation of an alternative autophagic pathway in macrophages at later stages of infection, the classical pathway was found to be inhibited at both early and later infection time points (58). These findings imply that, in the complex and dynamic relationship that exists between host cell autophagy and specific *Leishmania* parasite species, it is possible that cellular autophagy is regulated both during the establishment of infection as well as along the course of infection.

### Autophagic Features Present in *Leishmania*-Induced Parasitophorous Vacuoles

In addition to LC3, several other molecules have been explored as soluble markers for monitoring autophagy in vacuoles, including monodansylcadaverine (MDC) (98), acridine orange (99), neutral red (100), DQ-BSA (59), LysoSensor (101), and LysoTracker (102). Since LC3-labeling in compartments was shown to positively correlate with the number of autophagosomes in mammalian cells, the quantification of this marker has been proven to be a useful tool in studies investigating the participation of autophagy in diverse range of cellular processes (103).

To date, few studies have characterized *Leishmania*-induced parasitophorous vacuoles using these markers (59, 83). A comparative study employing CBA mouse macrophages found no significant differences between the percentage of *L. amazonensis*- or *L. major*-induced PVs positive for the acidic LysoTracker marker (59). Instead of quantifying the frequency of vacuoles expressing Lysotracker positivity, Real and Mortara (83) determined the fluorescence intensity of this probe in vacuoles induced by *L. amazonensis* and *L. major*. These authors demonstrated that the PVs induced by *L. major* presented less intense Lysotracker labeling than those induced by *L. amazonensis*. It is possible that the differences in LysoTracker assessment reported by Real and Mortara (83) and Dias et al. (59) may be due to divergent experimental designs, i.e., the animal models employed and the techniques used to characterize Lysotracker labeling. Our group also compared the hydrolytic activity of *L. amazonensis*- and *L. major*-induced PVs using DQ-BSA, a degradative compartment marker. The enhanced hydrolytic activity seen in *L. major* PVs compared to *L. amazonensis* seems to indicate greater degradative activity in the PVs induced by *L. major*, as evidenced by increased DQ-BSA dequenching (Figure 2). This finding led us to speculate that the lower degradative activity seen in *L. amazonensis*-induced PVs could favor parasite survival and multiplication within infected macrophages (59).

In addition to comparing the labeling of soluble markers in parasitophorous vacuoles induced by *L. amazonensis* and *L. major*, we compared the recruitment of LC3 to PVs induced by these *Leishmania* species. After 30 min of infection, the percentage of LC3-positive PVs was similar in cells infected by *L. major* and *L. amazonensis*. However, after 4 and 24 h, higher LC3 positivity was observed in *L. amazonensis*-induced PVs than in *L. major* [Figure 2; (59)]. Interestingly, the degree of LC3 recruitment to *L. amazonensis*- or *L. major*-induced PVs remained unchanged after treatment with an autophagic inhibitor, VPS34-IN1, or with the autophagic inducer, rapamycin (Figure 2). Similarly, Thomas et al. (58) found that autophagy induced by rapamycin did not modify LC3 labeling in *L. donovani*-infected THP-1 cells in comparison to untreated cells. It is possible that in both the Thomas et al. (58) and Dias et al. (59) studies, *Leishmania* was able to induce autophagic activation via a pathway other than PI3K-Akt-mTOR.

Although it has been shown that *Leishmania*-induced PVs present autophagosomal features, the internalization of parasites within autophagosomes has not been clearly observed, as was demonstrated for some bacteria, such as *M. tuberculosis* (104) and *Pseudomonas aeruginosa* (105). Although Frank et al. (95) used TEM to demonstrate interactions between myelin-like structures and the parasite plasma membrane, these authors did not report the complete engulfment of *L. major* in autophagosomes.

### Modulation of Autophagy and Influence on *Leishmania* Infection

#### In vitro

The effects on *Leishmania* infection outcome arising from the modulation of autophagy deserve more comprehensive study. Pinheiro et al. (55) demonstrated that the physiological induction of autophagy produced specific effects in *L. amazonensis* infection depending on the strain of mouse macrophage. In susceptible BALB/c macrophages, but not in resistant C57BL/6, the induction of autophagy enhanced *L. amazonensis* intracellular viability [Figure 3; (55)]. In addition, these authors showed that physiologically induced autophagy did not alter intracellular *L. major* parasite load in BALB/c mice macrophages [Figure 3; (55)]. Using the CBA mouse model, which controls *L. major* infection but is susceptible to *L. amazonensis*, we found that while the inhibition of autophagy did not affect *L. amazonensis* or *L. major* intracellular viability, pharmacologically- and physiologically-induced autophagy did increase intracellular viability in both species (Figure 3). More interestingly, we demonstrated greatly increased intracellular viability secondary to autophagic induction in *L. major* infection, in which vacuoles exhibited more degradative features (59).

Thomas et al. (58) demonstrated that the knockdown of Atg5 and Atg9 in THP-1 cells leads to reductions in intracellular *L. donovani* survival (Figure 3). On the other hand, Atg5 knockdown in BALB/c (95) and C57BL/6 (57) macrophages increased *L. major* parasitic load (Figure 3). Studies reporting that autophagic modulation inhibits *Leishmania* (88) or *Mycobacterium* phagocytosis (89) support the notion that by using a genetic approach to inhibit autophagy prior to infection, the phagocytic capacity of macrophages becomes affected as opposed to intracellular pathogen survival. Thus, a sound approach to studying the effects of modulating autophagy-related genes in the context of *Leishmania* infection is to transfect cells after infection, or to use plasmids with inducible promoters.

#### In vivo

To date, only one study has evaluated the effects of autophagic modulation on *Leishmania* infection *in vivo*. Franco et al. (57) demonstrated that intraperitoneal treatment with rapamycin for 10 days reduced ear lesion size by approximately 50% compared to control animals treated with ethanol, the drug vehicle. However, no alterations in parasitic load at lesion sites or in draining lymph nodes were seen in response to this treatment. The authors suggested that more prolonged treatment with rapamycin may be necessary to reduce parasite replication (57).

### Role of Autophagic Modulation in Proinflammatory Molecule Production by *Leishmania*-Infected Macrophages

To clarify how the exogenous induction of autophagy favors the intracellular viability of *L. amazonensis* and *L. major*, our group evaluated NO production and arginase activity in infected CBA macrophages. Although we found that exogenously induced autophagy decreases NO levels in both *L. major*- and *L. amazonensis*-infected macrophages, no differences in arginase activity were detected (59). Similarly, Pinheiro et al. (55) demonstrated that the physiological induction of autophagy decreased NO production by *L. amazonensis*-infected macrophages in association with increased intracellular parasite viability. Activation of the autophagic pathway was also shown to reduce NO production by RAW 264.7 macrophagic cells (106) and microglia (107), suggesting that autophagic effects on NO production seem to be universal.

In addition to evaluating NO production and arginase activity, Pinheiro et al. (55) investigated other key elements of the inflammatory response, including TGF-β, prostaglandin E2 (PGE2) and lipid body formation. Starvation was not shown to alter TGF-β production by infected macrophages, suggesting that decreased NO production occurs independently of TGF-β production. Lipid bodies are dynamic cytoplasmic organelles involved in lipid metabolism, membrane and vesicular transport and cell signaling (108). PGE2, an eicosanoid derived from the metabolism of arachidonic acid (AA) by the cyclooxygenase enzyme, is primarily produced in this organelle. It was previously demonstrated that PGE2 increased *Leishmania* intracellular viability (109, 110). Pinheiro et al. (55) showed that autophagic induction increased the production of both lipid bodies and PGE2 in *L. amazonensis-*infected BALB/c macrophages. Interestingly, in macrophages that were not submitted to the exogenous induction of autophagy, the addition of PGE2 enhanced *L. amazonensis* intracellular viability. Correspondingly, starvation-induced autophagy failed to increase parasitic load in infected BALB/c macrophages, which had also been treated with indomethacin, a cyclooxygenase inhibitor (55). These results suggest that the physiological induction of autophagy favors *L. amazonensis* intracellular viability by way of a mechanism related to enhancements in lipid body and PGE2 production, in addition to reduced levels of NO.

### Metabolic Regulation of Cellular Autophagy During Infection

Some studies in host cells have indicated that *Leishmania* modulates metabolic processes, including the metabolism of arginine, iron and lipids, in an attempt to generate a more permissive environment for survival (111). To the best of our knowledge, no studies have addressed the effects of autophagic modulation during *Leishmania* infection on host metabolism. As mentioned above, it has already been demonstrated that the induction of autophagy reduces NO production in *L. amazonensis*- and *L. major*-infected macrophages (55, 59). In host macrophages, NO is produced from the oxidation of L-arginine by inducible nitric oxide synthase (iNOS), thereby contributing to parasite killing (112, 113). On the contrary, arginase hydrolyzes L-arginine, producing ornithine and urea, which provide polyamines to the host cell, resulting in the blocking of NO production that can support parasite proliferation (114). Since both arginase and iNOS have L-arginine as a common substrate (114, 115), it is plausible to propose that the induction of autophagy, in addition to decreasing NO production (55, 59), may provide polyamines to *Leishmania*-induced vacuoles, which would subsequently favor the growth and intracellular development of *Leishmania* spp. Reinforcing this idea, a previous microarray study demonstrated that genes involved in arginine metabolism are upregulated in *L. amazonensis*-infected bone marrow macrophages (116). Contradictory to these findings, in CBA macrophages infected with *L. amazonensis* or *L. major*, in which autophagy has been induced, the reduction in NO production was not found associated with an enhancement in arginase production (59).

The ion metallic element, iron, shown to be important in mammalian and unicellular organisms, can be mobilized through autophagy from its cytosolic source, ferritin (117, 118). Then, ferrous iron can be released into *Leishmania*-induced PVs by way of an unknown transporter. Subsequently, iron reaches leishmanial cytosol through the LIT1 transporter present in the plasma membrane of intracellular amastigotes (119).

It has been demonstrated that regardless of *Leishmania* infection, autophagic induction can modulate lipid metabolism in mammalian cells (120, 121). It is possible that macrophage lipids may not only be a source of nutrients for amastigotes, but also could contribute to the biogenesis of PVs. Of note, Osorio y Fortea et al. (116) found that genes involved in lipid metabolism are upregulated in *L. amazonensis*-infected macrophages, which suggests that this parasite exploits host cell sterol biosynthesis machinery for sterol-dependent remodeling and the expansion of PV membranes. Further experimentation is required to determine whether autophagy is involved in this parasite-induced modulation of host lipid metabolism. Nonetheless, Singh et al. (120) demonstrated that under starvation conditions triglycerides can be mobilized as a result of lipid droplet degradation via the activation of selective autophagy in mammalian cells, but not infected macrophages. In sum, further studies must endeavor to investigate the triangularity of connections among autophagy, cellular metabolism, and *Leishmania* infection, in order to provide insight and further the development of more specific therapeutic targets for the control of *Leishmania* infection.

## Concluding Remarks

The relevance of the autophagic pathway in *Leishmania* infection remains poorly understood. The present review endeavors to summarize the current knowledge surrounding the importance of autophagy in *Leishmania* spp. infection. Several investigations have consistently demonstrated the induction of autophagy by all of the *Leishmania* spp. analyzed, as evidenced by enhanced LC3 labeling *in vitro* and *in vivo* (56–59, 92, 94–96). However, only two of these studies attempted to evaluate LC3 labeling in the membranes of parasite-induced PVs, and both found vacuoles decorated by LC3 in the context of *L. amazonensis* and *L. major* infection (59, 92). Furthermore, it remains unclear how LC3 is recruited to PVs. Meanwhile, although published data seem to support the notion that *L. donovani* inhibits canonical autophagy, this species has been reported to alternatively activate the autophagic pathway; however, the mechanism underlying this activation requires further clarification. Importantly, the scarce investigative studies that have attempted to evaluate how autophagic activation influences the pathogenesis of *Leishmania* infection have produced both variable and inconclusive results, which seem to be highly dependent on host cell background and/or parasite species. Thus, given the complexity of *Leishmania*-host interaction, important next steps should include the pursuit of autophagic pathway modulation using genetic approaches. Finally, the following open questions should be addressed: Do *Leishmania* spp. induce autophagy differently depending on host cell background? What are the mechanisms involved in *Leishmania*-induced autophagy? Exactly what role does autophagy play in *Leishmania* infection outcome? How can we untangle the complex associations between autophagy, cellular metabolism, and *Leishmania* infection? The answers to these queries will greatly enhance our understanding of how autophagy participates in *Leishmania* infection, and will permit the incorporation of relevant knowledge into the development of therapeutic strategies, including the modulation of specific autophagic pathways.

## Author Contributions

BD, JM, and PV contributed to manuscript elaboration and revision and approved the final version prior to submission.

### Conflict of Interest

The authors declare that the research was conducted in the absence of any commercial or financial relationships that could be construed as a potential conflict of interest.
